# Examining patient flow in a tertiary hospital’s emergency department at a low coronavirus prevalence region

**DOI:** 10.1186/s12873-022-00694-6

**Published:** 2022-07-27

**Authors:** Wen-Min Tseng, Po-Hsiang Lin, Pin-Chieh Wu, Chih-Hsiang Kao

**Affiliations:** 1 Department of Emergency Medicine, Kaohsiung Veterans General Hospital, No.386, Dazhong 1st Rd., Zuoying Dist, Kaohsiung City, 813414 Taiwan; 2 Health Management Center, Kaohsiung Veterans General Hospital, Kaohsiung City, Taiwan; 3 Department of Family Medicine, Kaohsiung Veterans General Hospital, Kaohsiung City, Taiwan; 4 Department of Nursing, Meiho University, Pingtung County, Taiwan

**Keywords:** COVID-19 pandemic, Low prevalence, Patient flow, Emergency department, Overcrowding

## Abstract

**Background:**

Taiwan’s successful containment of the COVID-19 outbreak prior to 2021 provided a unique environment for the surveillance of unnecessary emergency medical use. The aim of the study is to examine the impact of the coronavirus disease (COVID-19) pandemic on the patient flow in the emergency department (ED) of a tertiary hospital over 1 year in southern Taiwan, a region with low COVID-19 prevalence.

**Methods:**

Cross-sectional observational study was conducted from January to December 2020. Essential parameters of patient flow in the ED between January and February 2020 and the subsequent 11-month period were compared to data from 2019. Data were analyzed with descriptive statistics, using an independent sample *t*-test or Mann–Whitney U test, as applicable.

**Results:**

The ED census showed an acute decline (− 30.8%) from January to February 2020, reaching its nadir (− 40.5%) in April 2020. From February to December 2020, there was an average decrease of 20.3% in ED attendance (*p* < 0.001). The impact was most significant in ambulatory visits, lower-urgency acuity (level III) visits, and pediatric visits, without change in the acuity proportion. The length of stay shortened mainly in the adult division, which typically had an overcrowding problem (median, 5.7–4.4 hours in discharge; 24.8–16.9 hours in hospitalization; *p* < 0.001). The incidence of 72-hour unscheduled return visits was also reduced (4.1–3.5%, *p* = 0.002).

**Conclusions:**

In contrast to devastated regions, the impact on the ED patient flow in regions having low COVID-19 prevalence highlights a remodeling process of emergency medical care that would improve overcrowding.

**Supplementary Information:**

The online version contains supplementary material available at 10.1186/s12873-022-00694-6.

## Background

During the past two decades, coronavirus infections, known as severe acute respiratory syndrome (SARS) coronavirus (CoV) and Middle East respiratory syndrome (MERS) CoV, have been a subject of public health concern [1] and have affected the patient flow in hospital emergency departments (ED) [2–7]. SARS-CoV was the first major novel infectious disease to impact the international community in the twenty-first century [8], causing a total of 181 fatalities among 668 probable cases between March and July, 2003 in Taiwan, ranking it third globally [9, 10]. SARS-CoV-2 (hereafter COVID-19) has overwhelmingly surpassed SARS and MERS, both in terms of the number of infected people and epidemic areas [1] since 2019. Learning from the SARS experience, Taiwan’s successful containment of the COVID-19 outbreak prior to 2021 was achieved by effective mask wearing, contact tracing, and border control rather than a national lockdown [11, 12]. Since the first imported case from China on January 21, 2020 [13], there was only a limited local outbreak of COVID-19 infections in March 2020 [14]. At the end of 2020, the prevalence of the COVID-19 pandemic in Taiwan remained low, at 34 confirmed COVID-19 cases per million people [15], most of which were imported through international travellers, whereas indigenous cases were common in northern Taiwan [14, 16, 17]. Meanwhile, the global prevalence is high, with an estimate of over 60,000 cases per million people in the United States, 35,000 in Brazil, 34,000 in the European Union, and 7000 in India [15].

Patient flow is the movement of patients through a healthcare facility. Identifying changes in the pattern of patient flow in the ED is important for preparedness and mitigation. During the early days of the COVID-19 pandemic, a characteristic change and significant decrease in the number of patients who came to the ED with complaints unrelated to COVID-19 was reported [18–29], mostly in devastated regions, limited sections, or only for a short period of observation. However, the impact of patient flow throughout the pandemic year on the ED of a tertiary hospital, in a low-prevalence region, has not been comprehensively examined, especially in a health system that from April to December 2020 (i.e., 253 days) had no locally transmitted cases reported [30]. Because decreases in ED visits in severe pandemic areas were soon overcome by a surge in the numbers of COVID-19 admitted cases, attempts to distinguish between COVID-19 and ED visits by patients seeking care for reasons unrelated to COVID was difficult to obtain [19, 22, 31]. In contrast, observations from our hospital, where we did not experience a surge in COVID-19 infection throughout 2020, help to clarify the characteristics of the patient flow of an ED that were unrelated to COVID-19 infection. Changes in these characteristics after entering the era of the pandemic as well as the impacts of such changes on ED crowding warrant detailed investigations.

This study aimed to examine how essential parameters of patient flow changed in the ED of a tertiary hospital, without diagnosis of any indigenous COVID-19 cases from visits in 2020 compared to the same period in 2019. We reviewed the changes in patient flow from the initial to the late phase of the pandemic and the effects of this unique circumstance on ED crowding. We also attempted to compare these changes between a low-prevalence region and the devastated areas as well as between the COVID-19 pandemic and the SARS epidemic. These findings may provide valuable guidance for reducing unnecessary ED attendance and the allocation of emergency medical resources for the next novel virus pandemic.

## Methods

### Study design, setting, and selection of participants

A retrospective, observational, cross-sectional study of patient flow from January 2019 to December 2020 in an ED was conducted at Kaohsiung Veterans Hospital (KSVGH), a 1482-bed tertiary medical center located in a metropolitan area of southern Taiwan with an average of 86,496 annual ED visits (95% confidence interval [CI], ±1666) from 2009 to 2019 before the COVID-19 pandemic. The KSVGH is a level I trauma center with all surgical subspecialties available 24 h a day. In our ED, divisions of care are categorized into adult, trauma, pediatric (aged < 18 years), and others (e.g., emergency dentistry, psychiatry, delivery). We included all patients who were assessed by emergency physicians with a medical record in our ED from 2019 to 2020, while those who were discharged from the ED without being evaluated or those who were referred to outpatient department without receiving treatment or survey were excluded from the current study.

### Measurements

We examined a cross-section of patient flow variables in the initial epidemic period between January 2020 (referred to as P1) and February 2020 (referred to as P2), and the subsequent period from February to December 2020, referred to as the post-epidemic period (P20), compared to the same period in 2019, referred to as the pre-epidemic period (P19). We obtained the demographics, visit characteristics, divisions of care, arrival modes, and acuity levels as input factors; door-to-doctor time (D2d) and length of stay (LOS) as throughput factors; and disposition decisions and adverse consequences of the patients as output factors. Patient characteristics include age, gender, divisions of care, arrival modes, and acuity levels. Ages were divided into four subgroups as young children (< 5 years), children (5**–**17 years), adults (18**–**64 years) and the elderly(≥ 65 years). Triage at the ED divided all patients into four categories based on their age and the nature of their problems, namely, adults, trauma, pediatrics, and others. All patients regardless of age who visited the ED because of trauma belonged to the category of “trauma”, while others fell into the categories of “adults” and “pediatrics” at a cutoff point of 18 years of age. On the other hand, patients who required care by specialists (e.g., emergency dentistry, psychiatry, and obstetrics) were categorized as “others”. The arrival modes of patients to the ED are classified into ambulation, transportation by emergency medicine service (EMS), and transfer-in from another hospital. We considered out-of-hospital cardiac arrest (OHCA) as an isolated index to monitor an undiscovered epidemic or public reluctance to visit hospitals. In addition, patients who arrived at the ED by ambulance fell into the “emergency medicine service” (EMS) group, while those with a transfer sheet from other hospitals or clinics were assigned to the “transfer-in” group. All other patients were defined as “ambulatory”. The acuity level was classified using the 5-level Taiwan Triage Acuity System, with level I being the highest urgent need and level V the least urgent need.

The LOS was measured from the time of ED registry to the departure of either admitted or discharged patients in the adult, trauma, and pediatric divisions. Patients categorized under the “other” group were excluded because they accounted for < 1% of the total ED volume. D2d was the waiting time from registration at the ED to diagnostic evaluation by a physician. The D2d of acuity level I was excluded since these patients were immediately presented to the resuscitation bay, where a physician is always present, making the D2d time nearly zero. Disposition decisions included discharge, hospitalization, transfer-out, discharge against medical advice (DAMA) and deceased (discharge either upon death or impending death). These parameters were essential data that needed to be collected by the physician-in-charge and documented in the discharge notes. Adverse outcomes included in-hospital cardiac arrest (IHCA) and unscheduled return visits (URVs) within 72 h.

### Data collection

Preliminary datasets covering all patients eligible for the current study (i.e., adults, trauma, pediatrics, and others) from ED registration to their discharge from ED were retrieved from the hospital information system after the process of patient de-identification. Despite successful refilling of all missing parameters and data according to the information available in the database after manual rechecking of the medical records, we identified 69 missing items on the monthly average of the mode of arrival that accounted for 1.03% of the total number of ED visits. To minimize the impact of those missing data on our analysis and to achieve a comparable numbers of patients in each category, we pooled the missing data to the ambulatory group that accounted for the majority of missing data. This study was reviewed and approved by the institutional review board of the KSVGH (KSVGH21-CT5–16) that waived the requirement for an informed consent from the patients in accordance with the relevant guidelines and regulations.

### Data analysis

All digital data were automatically extracted from the electronic medical record system through the application of appropriate filters. and analyzed using the SPSS software v.20.0 (SPSS Inc., Armonk, NY, USA). We estimated the numbers and ratio changes of variables in the initial epidemic period between P1 and P2 and the subsequent period in P20 as a historical comparison to P19. Data on daily ED visits and patients’ LOS are displayed as moving average for comparison between 2019 and 2020. The variables were compared using an independent samples t-test or Mann–Whitney U test as appropriate. All statistical tests were two-sided, and we considered a result to be significant when the *P*-value was < 0.05.

## Results

**Fig. 1:**
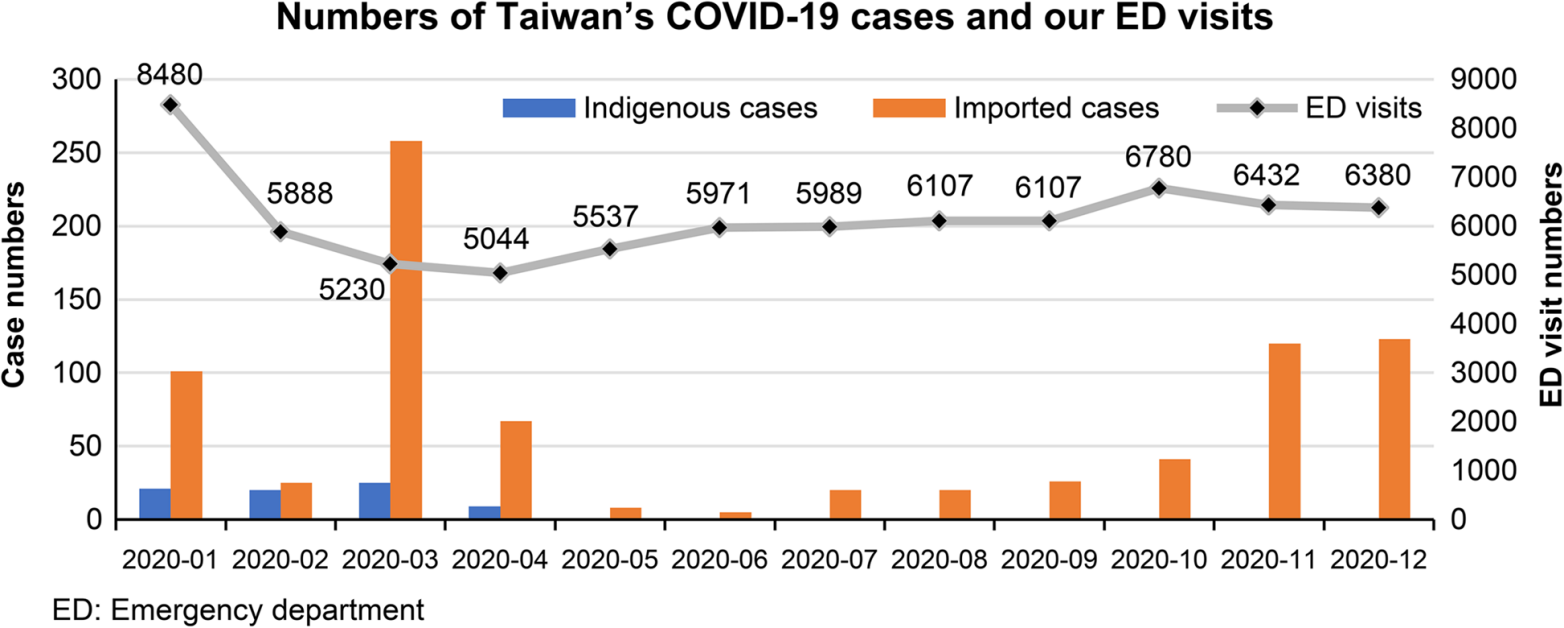
Taiwan’s monthly numbers of COVID-19 cases and visits to the Kaohsiung Veterans General Hospital emergency department in 2020

Of a total of 166,687 visits to the ED from 2019 to 2020, 2267 (1.4%) were excluded (i.e., 1351 in 2019 and 916 in 2020). Patient demographics, input, throughput, and output factors of the ED during the four periods are summarized in Tables 1, 2 and 3, respectively. Total visits to the ED declined from 8486 in January 2020 (P1) to 5871 (− 30.8%, *p* < 0.001) in February 2020 (P2), and the number of visits reached its nadir at 5044 (− 40.5%, *p* < 0.001) in April 2020 (Fig. 1). Among different age groups, the number of visits decreased to varying degrees. There was a disproportional decrease between children and adults, from 57.8%(*p* < 0.001) in young children, 50.8%(*p* < 0.001) in children, to 25.9%(*p* < 0.001) in adults, and 20.8%(*p* < 0.001) in the elderly. Likewise, the visit numbers decreased more significantly in the pediatric group (1575 vs. 666, − 57.7%, *p* < 0.001), whereas the degree was similar between adults overall (5413 vs. 4078, − 24.7%, *p* < 0.001) and the trauma section (1417 vs. 1089, − 23.1%, *p* < 0.001). For both acuities levels II and III, which accounted for over 90% of ED visits, the decrease was − 29.1% (1532 vs. 1086, *p* < 0.001) and − 32.8% (6489 vs. 4358, *p* < 0.001), respectively. The number of patients with acuity level I, as the highest urgency group, showed no difference (250 vs. 243, *p* = 0.68), which was similar to patients with OHCA (15 vs. 16, *p* = 0.71). The arrival modes to the ED decreased most significantly in ambulation (7077 vs. 4517, − 36.2%) followed by transfer-in (630 vs. 588, − 6.7%) and transported by EMS (779 vs. 766, − 1.7%). The decrease of D2d time was most significant in acuity level III (*p* < 0.001). The LOS regarding discharge and hospitalization had no significant difference, except for pediatric patients who were hospitalized (median, 4.1 h vs. 3.0 h; *p* = 0.015). The boarded bed count of the whole hospital increased 2.5% (from 3843 to 3939). The reduction rate in the number of discharged patients (6516 vs. 4257, − 34.7%, *p* < 0.001) was higher than that of hospitalized patients (1687 vs. 1340, − 20.6%, *p* < 0.001). The proportion of patients with DAMA (2.7% vs. 3.6%, *p* = 0.82), death (0.4% vs. 0.7%, *p* = 0.33), IHCA (0.21% vs. 0.39%), and URVs (3.38% vs. 3.41%) increased slightly.

**Table 1:** Differences in the number of emergency department visits and the proportion in each category between the initial period and the subsequent period

	2020 Jan No. (%)	2020 Feb No. (%)	% Change	2019 Feb–Dec No. (%)	2020 Feb–Dec No. (%)	% Change	***p***–value	
	[daily mean, 95%CI]	[daily mean, 95%CI]	[daily MD, 95%CI]	[daily mean, 95%CI]	[daily mean, 95%CI]	[daily MD, 95%CI]	(No.)	(%)
**Total No. of ED visits**	8486 (100)	5871 (100)	−30.8%	82,049 (100)	65,412 (100)	−20.3%	< 0.001*	
	[273.7, 248.7–298.8]	[202.4, 194.3–210.6]	[71.3, 43.6–98.9]	[245.7, 241.3–250.0]	[195.3, 192.3–198.2]	[50.4, 45.1–55.7]		
**Age**								
**< 5**	950 (11.2)	401 (6.8)	−57.8%	9537 (11.6)	5643 (8.6)	−40.8%	< 0.001*	< 0.001*
				[28.6, 27.5–29.7]	[16.8, 16.0–17.7]	[11.7, 10.3–13.1]		
**5–17**	898 (10.6)	442 (8)	−50.8%	7414 (9.0)	4329 (6.6)	−41.6%	< 0.001*	< 0.001*
				[22.2, 21.3–23.0]	[12.9, 12.4–13.4]	[9.3, 8.3–10.3]		
**18–64**	4476 (52.7)	3315 (56)	−25.9%	42,175 (51.4)	36,159 (55.3)	−14.3%	< 0.001*	< 0.001*
				[126.3, 123.8–128.7]	[107.9, 106.2–109.7]	[18.3, 15.3–21.4]		
**≥ 65**	2162 (25.5)	1713 (29.2)	−20.8%	22,923 (27.9)	19,281 (29.5)	−15.9%	< 0.001*	< 0.001*
				[68.6, 67.6–69.6]	[57.6, 56.6–58.6]	[11.1, 9.7–12.5]		
**Gender**								
**Female**	4210 (49.6)	2782 (47.4)	−33.9%	38,870 (47.4)	31,256 (47.8)	−19.6%	< 0.001*	0.117
				[116.4, 114.0–118.7]	[93.3, 91.6–95.0]	[23.1, 20.2–26.0]		
**Male**	4276 (50.4)	3089 (52.6)	−27.8%	43,179 (52.6)	34,156 (52.2)	−20.9%	< 0.001*	0.117
				[129.3, 126.9–131.6]	[102.0, 100.2–103.7]	[27.3, 24.4–30.2]		
**Division**								
**Adults**	5413 (63.8)	4078 (69.5)	−24.7%	52,206 (63.6)	43,061 (65.8)	−17.5%	< 0.001*	< 0.001*
				[156.3, 153.5–159.1]	[128.5, 126.6–130.5]	[27.8, 24.4–31.2]		
**Trauma**	1417 (16.7)	1089 (18.5)	−23.1%	15,275 (18.6)	14,140 (21.6)	−7.4%	< 0.001*	< 0.001*
				[45.7, 44.9–46.6]	[42.2, 41.3–43.1]	[3.5, 2.3–4.8]		
**Pediatrics**	1575 (18.6)	666 (11.3)	−57.7%	14,442 (17.6)	7713 (11.8)	−46.6%	< 0.001*	< 0.001*
				[43.2, 41.6–44.9]	[23.0, 21.9–24.1]	[20.2, 18.2–22.2]		
**Others**	81 (1.0)	38 (0.6)	−53.1%	126 (0.2)	498 (0.8)	295.2%	< 0.001*	< 0.001*
				[0.4, 0.2–0.5]	[1.5, 1.3–1.7]	[−1.1, −1.3 −−0.9]		
**Acuity (TTAS)**								
**I**	250 (2.9)	243 (4.1)	−2.8%	2739 (3.3)	2183 (3.3)	−20.3%	< 0.001*	0.949
				[8.2, 7.9–8.5]	[6.5, 6.2–6.8]	[1.7, 1.2–2.1]		
**II**	1532 (18.1)	1086 (18.5)	−29.1%	16,567 (20.2)	13,444 (20.6)	−18.9%	< 0.001*	0.742
				[49.6, 48.5–50.7]	[40.1, 39.1–41.2]	[9.5, 7.9–11.0]		
**III**	6489 (76.5)	4358 (74.2)	−32.8%	61,231 (74.6)	48,158 (73.6)	−21.4%	< 0.001*	0.050
				[183.3, 179.1–187.5]	[143.8, 141.2–146.3]	[39.6, 34.7–44.5]		
**IV**	152 (1.8)	149 (2.5)	−2.0%	958 (1.2)	1126 (1.7)	17.5%	0.023 *	< 0.001*
				[2.9, 2.6–3.2]	[3.4, 3.0–3.7]	[−0.5, −0.9–0.1]		
**V**	63 (0.7)	35 (0.6)	−44.4%	554 (0.7)	501 (0.8)	−9.6%	0.204	0.198
				[1.7, 1.5–1.8]	[1.5, 1.3–1.7]	[0.2, −0.1–0.4]		
**OHCA**	15 (0.2)	16 (0.3)	6.7%	165 (0.2)	182 (0.3)	10.3%	0.374	0.002*
				[0.5, 0.4–0.6]	[0.5, 0.5–0.6]	[0.0, −0.2–0.1]		
**Arrival Modes**	**2020 January No. (%)**	**2020 February No. (%)**	**% Change**	**2019 Feb–Dec Sum (%)**	**2020 Feb–Dec Sum (%)**	**% Change (Sum)**	***p–*value**	
**(MW U test)**				**(Median, P25–P75)**	**(Median, P25–P75)**		**(No.)**	**(%)**
**Ambulatory**	7077 (83.2)	4517 (77.0)	−36.2%	66,638 (81.2)	50,707 (77.5)	−24.9%	< 0.001*	< 0.001*
				(6065, 5924–6410.5)	(4624, 4372–4861.5)			
**EMS**	779 (9.4)	766 (13.0)	−1.7%	8318 (10.1)	8552 (13.1)	2.8%	0.178	< 0.001*
				(743, 722.5–777)	(796, 762.5–810.5)			
**Transfer-in**	630 (7.2)	588 (10.0)	−6.7%	7093 (8.6)	6153 (9.4)	−13.3%	< 0.001*	0.009*
				(644, 621–668.5)	(571, 532.5–588.5)			

*p < 0.05

*Abbreviations*: *No*. Number, *MD* Mean difference, *CI* Confidence interval, *Feb* February, *Dec* December, *ED* Emergency department, *TTAS* Taiwan Triage and Acuity Scale, *OHCA* Out-of-hospital cardiac arrest, *EMS* Emergency medical services, *MW U test* Mann–Whitney U test

**Table 2:** Differences in LOS and D2d in emergency department visits between the initial and subsequent period

	Jan 2020 Median (P25–P75)	Feb 2020 Median (P25–P75)	***p-***value	Feb–Dec 2019 Median (P25–P75)	Feb–Dec 2020 Median (P25–P75)	***p***-value
**LOS (hour)**						
**Discharge**						
**Total**	3.6 (3.0–4.4)	3.5 (3.1–4.1)	0.947	4.3 (3.7–5.1)	3.5 (3.1–4.1)	< 0.001*
**Adults**	4.6 (3.7–5.8)	4.3 (3.8–5.4)	0.620	5.7 (4.9–7.0)	4.4 (3.8–5.4)	< 0.001*
**Trauma**	2.0 (1.7–2.2)	1.8 (1.6–2.4)	0.947	1.9 (1.6–2.2)	1.9 (1.6–2.2)	0.229
**Pediatrics**	2.0 (1.7–2.2)	1.8 (1.4–2.1)	0.240	2.0 (1.7–2.4)	2.0 (1.6–2.4)	0.797
**Hospitalization**						
**Total**	13.6 (11.6–15.3)	14.6 (11.8–18.0)	0.108	19.1 (16.1–22.1)	14.2 (11.5–18.2)	< 0.001*
**Adults**	16.4 (13.7–18.5)	17.4 (14.4–21.6)	0.190	24.8 (20.7–28.5)	16.9 (13.6–22.2)	< 0.001*
**Trauma**	4.8 (3.4–5.6)	4.1 (3.4–5.5)	0.396	4.9 (4.0–6.2)	4.5 (3.8–6.1)	0.050
**Pediatrics**	4.1 (3.1–5.0)	3.0 (1.9–4.0)	0.015*	3.8 (3.0–5.1)	3.4 (2.3–5.1)	0.004*
**D2d (min: sec)**						
**Acuity** ** I**	**–**	**–**	**–**	**–**	**–**	–
**II**	04:58 (04:21–06:05)	04:38 (03:32–05:26)	0.122	04:52 (04:08–05:50)	04:10 (03:17–05:06)	< 0.001*
**III**	09:00 (08:16–10:31)	07:08 (06:14–08:07)	< 0.001*	08:16 (07:21–09:17)	07:13 (06:16–08:17)	< 0.001*
**IV**	10:32 (09:05–17:06)	08:46 (06:48–10:47)	0.003*	10:58 (06:58–15:43)	09:00 (06:12–12:29)	< 0.001*
**V**	09:45 (05:43–16:52)	12:15 (09:20–16:40)	0.454	09:05 (05:17–14:25)	08:05 (04:30–13:02)	0.163

*p < 0.05

*CI* Confidence interval, *Feb* February, *Jan* January, *LOS* Length of stay, *D2d* Door-to-door time, *min:sec* Minutes:seconds

*TTAS* Taiwan Triage and Acuity Scale

**Table 3:** Differences in disposition and adverse outcomes of emergency department visits between the initial and subsequent period

	2020 Jan No. (%)	2020 Feb No. (%)	% Change	2019 Feb–Dec No. (%)	2020 Feb–Dec No. (%)	% Change	***p***-value	
				[daily mean, 95%CI]	[daily mean, 95%CI]	[daily MD, 95%CI]	(No.)	(%)
**Disposition**	8486 (100)	5871 (100)	−30.8%	82,049 (100) [245.7, 241.3–250.0]	65,412 (100) [195.3, 192.3–198.2]	−20.3% [50.4, 45.1–55.7]	< 0.001*	
**Discharged**	6516 (76.8)	4257 (72.5)	−34.7%	60,802 (74.1)	46,315 (70.8)	−23.8%	< 0.001*	< 0.001*
				[182.0, 177.8–186.3]	[138.3, 135.5–141.0]	[43.8, 38.8–48.8]		
**Hospitalized**	1687 (19.9)	1340 (22.8)	−20.6%	18,265 (22.3)	16,581 (25.3)	−9.2%	< 0.001*	< 0.001*
				[54.7, 53.8–55.6]	[49.5, 48.5–50.5]	[5.2, 3.9–6.5]		
**Transferred**	21 (0.2)	25 (0.4)	19.0%	359 (0.4)	253 (0.4)	−29.5%	< 0.001*	0.109
				[1.1, 1.0–1.2]	[0.8, 0.7–0.8]	[0.3, 0.2–0.5]		
**DAMA**	230 (2.7)	210 (3.6)	−8.7%	2316 (2.8)	1947 (3.0)	−15.9%	< 0.001*	0.135
				[6.9, 6.6–7.3]	[5.8, 5.5–6.1]	[1.1, 0.7–1.6]		
**Died**	32 (0.4)	39 (0.7)	21.9%	307 (0.4)	316 (0.5)	2.9%	0.756	0.003*
				[0.9, 0.8–1.0]	[0.9, 0.8–1.1]	[0.0, −0.2–0.1]		
**MW U test**	**2020 Jan No. (%)**	**2020 Feb No. (%)**	**% Change**	**2019 Feb–Dec Sum (%)**	**2020 Feb–Dec Sum (%)**	**% Change (Sum)**	***p*-value**	
				**(Median, P25–P75)**	**(Median, P25–P75)**		**(No.)**	**(%)**
**Adverse Outcome**								
**IHCA**	18 (0.21)	23 (0.39)	27.8%	142 (0.17)	134 (0.20)	−5.6%	0.766	0.341
				(11, 9.5–17)	(12, 9.5–14)			
**URVs**	287 (3.38)	201 (3.41)	−30.0%	3355 (4.1)	2270 (3.5)	−32.3%	< 0.001*	0.002*
				(311, 274–332)	(209, 186–229)			
**Boarded Beds**	3843	3939	2.5%	47,817	46,500	−2.8%	0.291	
				(4410, 4339–4480)	(4327, 3969–4471)			

* *p* < 0.05

*Feb* February, *Dec* December, *MD* Mean difference, *CI* Confidence interval, *DAMA* discharges against medical advice, *IHCA* In–hospital cardiac arrest, *URVs* Unplanned return visits, *MW U test* Mann–Whitney U test

### Differences in ED visits between P19 and P20 overall, by age, and by hospital division

**Fig. 2:**
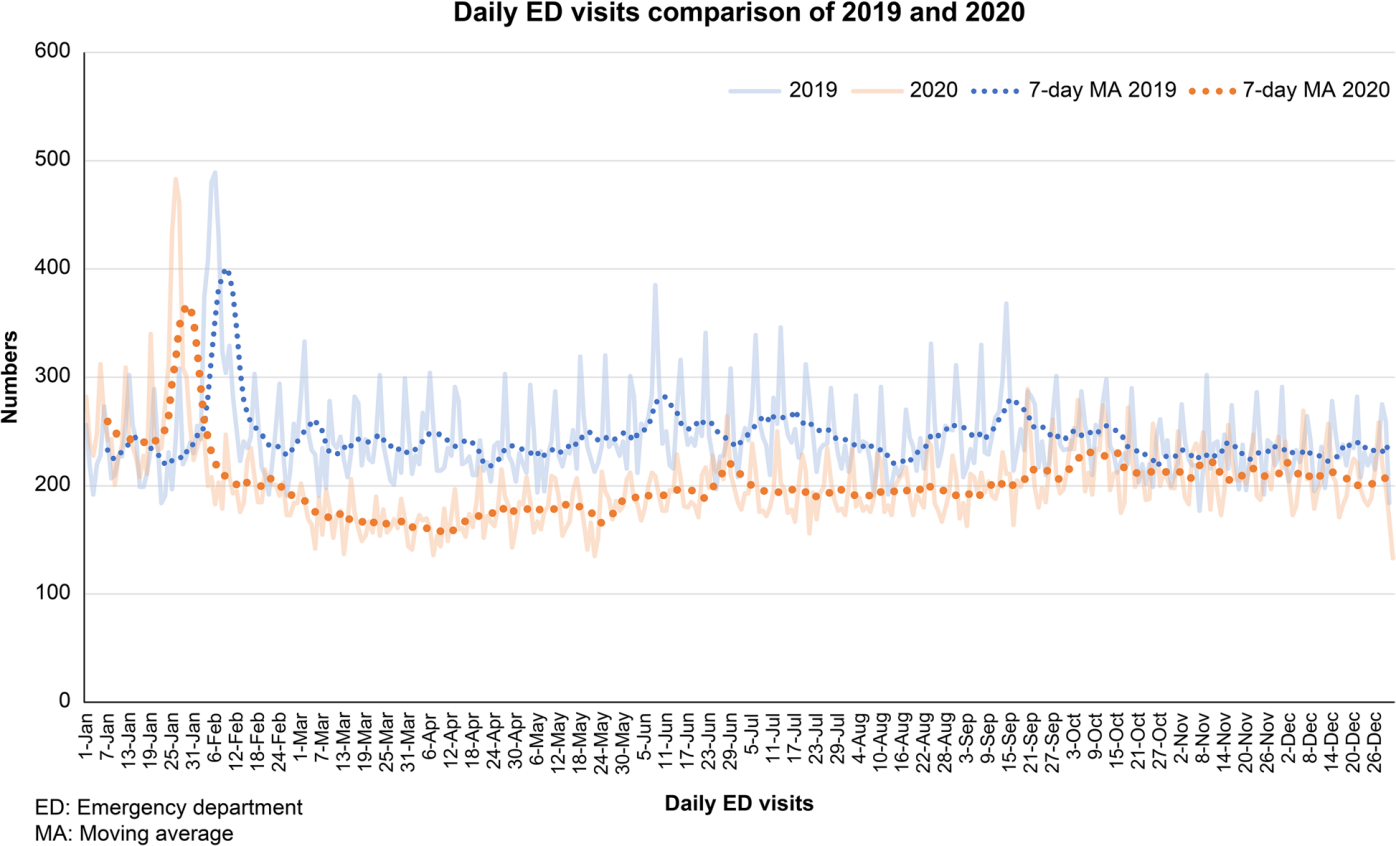
Comparison of daily visits to the Kaohsiung Veterans General Hospital emergency department between 2019 and 2020

**Fig. 3:**
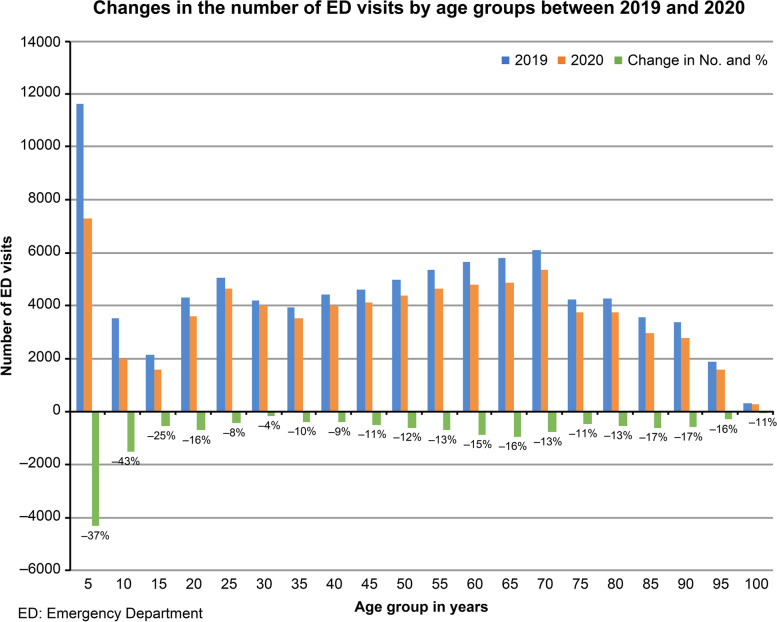
Changes in the number and percentage of visits by age groups to the Kaohsiung Veterans General Hospital emergency department between 2019 and 2020

**Fig. 4:**
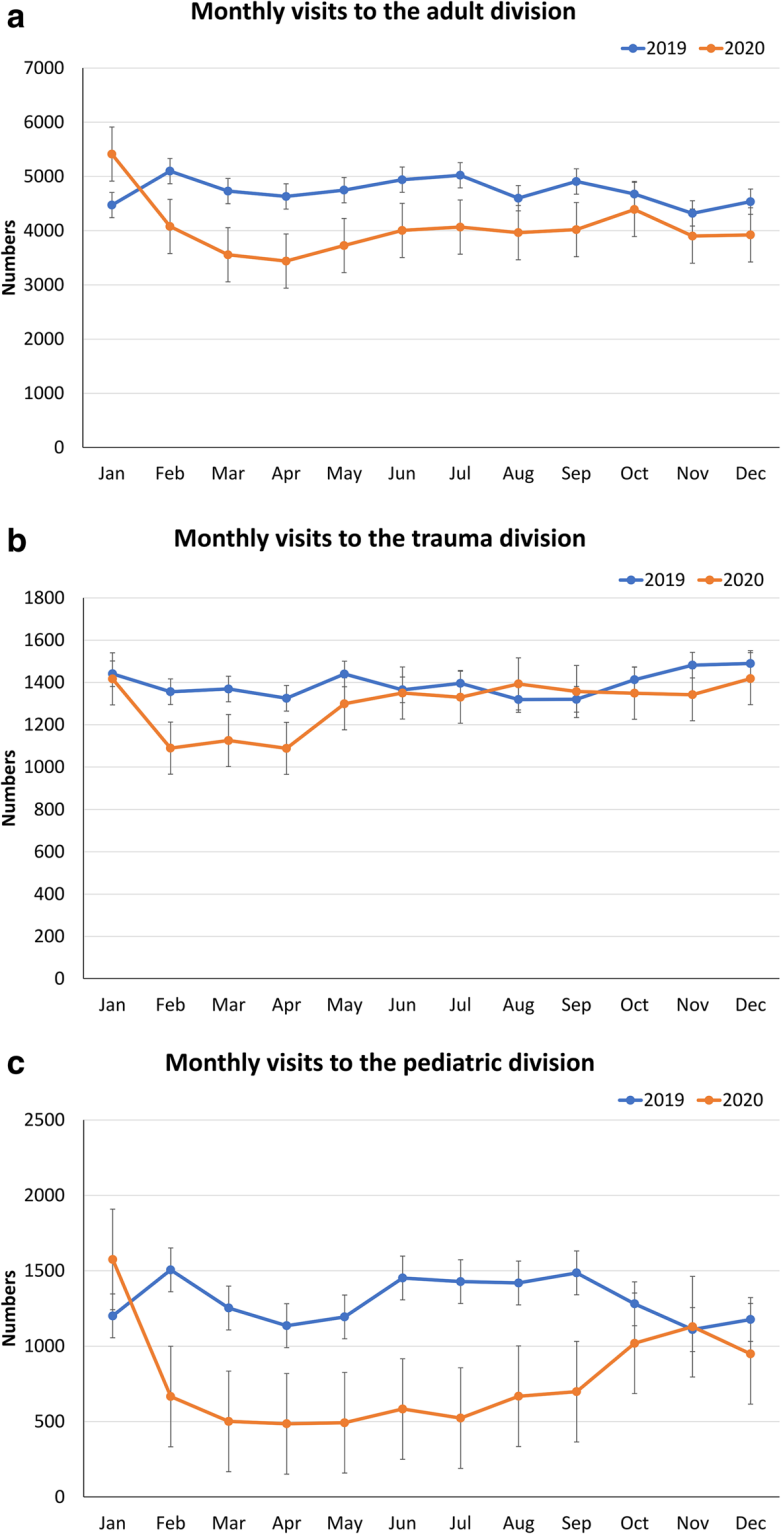
**a**. Comparison of monthly visits to adult division of the Kaohsiung Veterans General Hospital emergency department between 2019 and 2020. **b**. Comparison of monthly visits to trauma division of the Kaohsiung Veterans General Hospital emergency department between 2019 and 2020. **c**. Comparison of monthly visits to pediatric division of the Kaohsiung Veterans General Hospital emergency department between 2019 and 2020

The total number of ED visits decreased an average of 20.3%, from a daily mean of 245.7 to 195.3 (95% CI of daily mean difference [MD], 45.1–55.7; *p* < 0.001). A comparison of the daily ED census between 2019 and 2020 is shown in (Fig. 2). Among all age categories, the number of visits decreased to varying degrees and was inversely proportional to age, ranging from an average decrease of 40.8% (95% CI of daily MD, 10.3–13.1; *p* < 0.001) in young children, 41.6% (95% CI of daily MD, 8.3–10.3; *p* < 0.001) in children, 14.3% (95% CI of daily MD, 15.3–21.4; *p* < 0.001) in adults, and 15.9% (95% CI of daily MD, 9.7–12.5; *p* < 0.001) in the elderly (Fig. 3). The proportion of visits among children decreased, and, in contrast, the proportion of adult and elderly visits increased (*p* < 0.001 in all). The visits of each division decreased 17.5% (95% CI of daily MD, 24.4–31.2; *p* < 0.001) in adults (Fig. 4a), 7.4% (95% CI of daily MD, 2.3–4.8; *p* < 0.001) in trauma (Fig. 4b), and 46.6% (95% CI of daily MD, 18.2–22.2; *p* < 0.001) in pediatrics (Fig. 4c). Consistent with the age categories, the proportion of visit numbers in the pediatric division decreased, whereas the numbers in the adult and trauma divisions increased (*p* < 0.001 in all).

### Differences in the acuity of ED visits between P19 and P20

The encounters of acuity levels II and III, being the majority of ED attendance, decreased 18.9% (95% CI of daily MD, 7.9–11.0; *p* < 0.001) for level II and 21.4% (95% CI of daily MD, 34.7–44.5; *p* < 0.001) for level III. Among patients with the highest urgency (level I), the numbers surprisingly decreased to a similar degree (20.3%; 95% CI of daily MD, 1.2–2.1; *p* < 0.001). The proportion of visits in level I (*p* = 0.949), level II (*p* = 0.742), and level III (*p* = 0.05) did not change significantly, and that of level IV rose slightly from 1.2 to 1.7% (*p* = 0.023).

### Differences in patient arrival modes between P19 and P20

There was a significant difference in visit numbers in ambulatory and transferred patients (transfer-in), with a total 24.9% (median 6065 vs. 4624; *p* < 0.001) and 13.3% (median 644 vs. 571; *p* < 0.001) decrease, but there was no obvious change in those transported by EMS (median 743 vs. 796; *p* = 0.178). The proportion of patients arriving by ambulation decreased from 81.2 to 77.5% (*p* < 0.001) and increased from 10.1 to 13.1% for EMS (*p* < 0.001) and 8.6 to 9.4% for transfer-in (*p* = 0.009). The ratio of OHCA increased from 0.2 to 0.3% (*p* = 0.002) without a significant change in total numbers (165 vs. 182; *p* = 0.374).

### Differences in D2d and LOS between P19 and P20

**Fig. 5:**
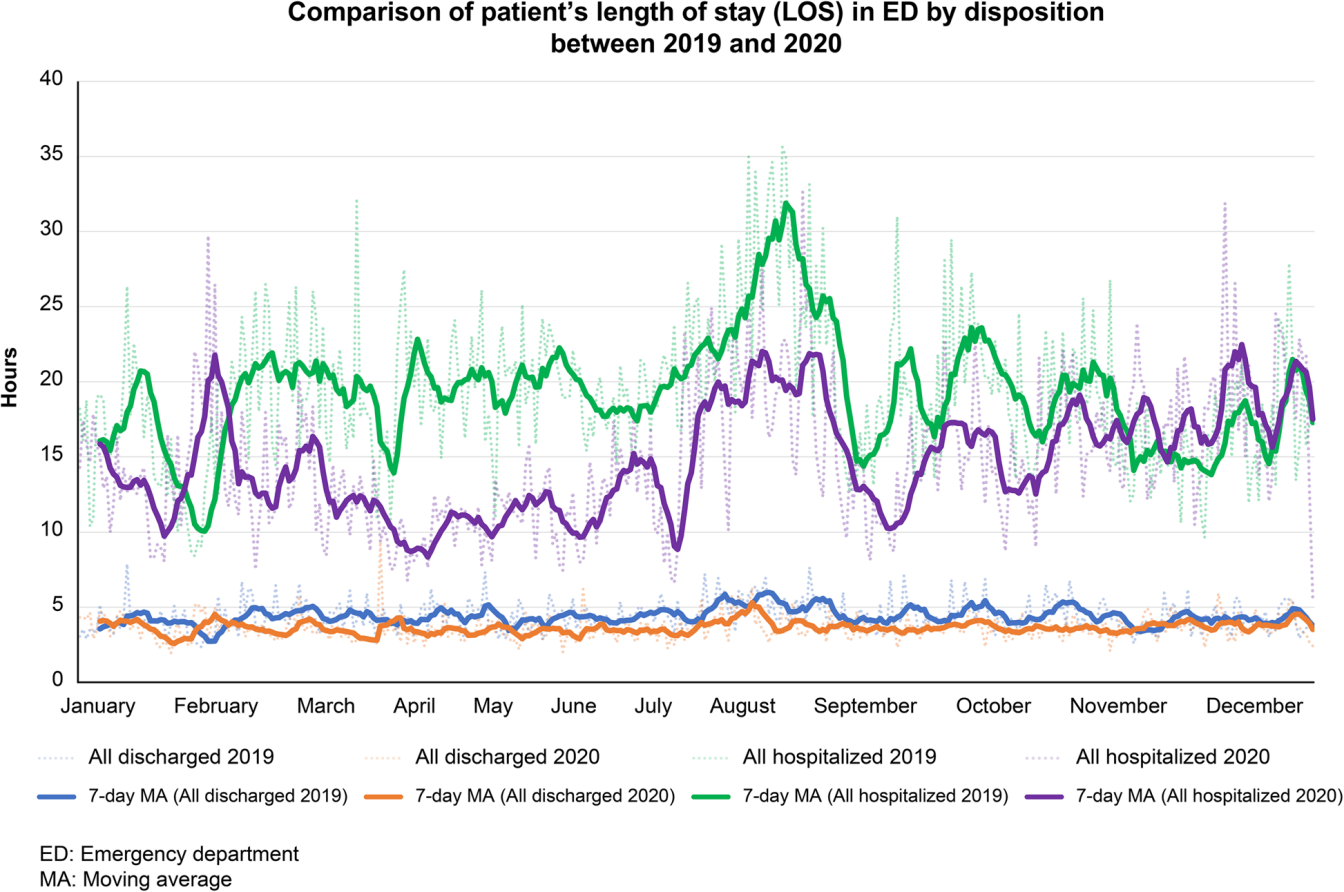
Comparison of length of stay of patients discharged or hospitalized from the Kaohsiung Veterans General Hospital emergency department between 2019 and 2020

The average D2d time of a patient at our ED seldom exceeds 10 min, so the difference between P19 and P20 was minimal, but it was significantly reduced (*p* < 0.001). The median LOS shortened from 4.3 to 3.5 h (*p* < 0.001) among discharged patients and 19.1 to 14.2 h (*p* < 0.001) among hospitalized patients (Fig. 5). By division, the median LOS of discharged patients shortened from 5.7 to 4.4 h in the adult division (*p* < 0.001), but there was no significant difference in the trauma division (*p* = 0.229) or the pediatric division (*p* = 0.797). The median LOS of hospitalized patients shortened significantly in all three divisions, from 24.8 to 16.9 h in the adult division (*p* < 0.001), 4.9 to 4.5 h in the trauma section (*p* = 0.05), and 3.8 to 3.4 h in the pediatric division (*p* = 0.004).

### Disposition changes between P19 and P20

The numbers of monthly boarded beds in the hospital did not change significantly (− 2.8%; median 4410 to 4327; *p* = 0.291) between P19 and P20. The average daily numbers decreased 23.8% (95% CI of daily MD, 38.8–48.8; *p* < 0.001) among discharged patients, 9.2% (95% CI of daily MD, 3.9–6.5; *p* < 0.001) among hospitalized patients, and 29.5% (95% CI of daily MD, 0.2–0.5; *p* < 0.001) and 15.9% (95% CI of daily MD, 0.7–1.6; *p* < 0.001) among patients who were transferred out and DAMA, respectively. The proportion of discharged patients dropped from 74.1 to 70.8% (*p* < 0.001), and the proportion of hospitalized patients rose from 22.3 to 25.3% (*p* < 0.001). The total number of deceased patients showed no significant changes (307 vs. 316; *p* = 0.756), with a slight increase from 0.4 to 0.5% (*p* = 0.003). There was no significant change in patients with IHCA (142 vs. 134; *p* = 0.766 and 0.17% vs. 0.20%; *p* = 0.341, for total number and percent of total patients, respectively) but a decrease in patients with 72-h URVs (3355 vs. 2270; *p* < 0.001 and 4.1% vs. 3.5%; *p* = 0.002, respectively).

## Discussions

### Impact of COVID-19 on ED attendance in the initial and later phase of the pandemic

The COVID-19 pandemic has posed a dramatic, unexpected, and unprecedented burden on many healthcare systems worldwide. Universally decreased numbers of ED visits by age, disease categories, and the characteristics and case severity of patients have been revealed by anecdotal reports [18–29, 31–36]. Most studies originated from severe pandemic areas that analyzed the change in the early stage (4–8 weeks) of the COVID-19 outbreak and reported a significant decrease, ranging from 31.0 to 63.5%, in ED patient flow compared to a historical control period [18, 19, 21, 23, 27, 31, 34]. During the initial phase, we found some similarities regarding a reduction in in ED visits, especially in pediatric and ambulatory patients, among the compared countries where the pandemic was serious [20, 21, 26, 27, 31]. In February 2020, most parameters used to measure the input of patient flow in our ED dropped sharply and reached their lowest point in April. Other tertiary hospitals and the largest healthcare organization in northern Taiwan found that ED visits decreased, similarly, from February to April 2020 [29, 33, 37].

In the later phase till the end of 2020, compared with the same period during the previous year, patient visits gradually recovered, but not completely. Throughout 2020, ambulatory visits, lower-urgency acuity (level III) cases, and the pediatric division still saw the greatest impact. The impact significantly disproportionate to age would correspond to the effect of these combinations. Compared with adults (Fig. 4a) and pediatrics (Fig. 4c), the trauma (Fig. 4b) division had the least reduction in the number of patients and recovered sooner, probably because there were few activity restrictions in Taiwan [12], and traumatic events usually have little to do with the spread of diseases.

### Possible reasons for the decline in ED attendance

Despite a low prevalence of COVID-19 in Taiwan, the visits to the ED declined significantly in all divisions to various degrees. This phenomenon could not be explained simply by lockdown and sports restrictions, which clearly led to the reduction of traumatic cases [22, 38], since restrictions were not strictly executed in Taiwan [12]. The reason why these patients did not visit the ED remains unclear; however, fear of contracting the virus, according to many observations worldwide, may be the cause [38–41], and this was also emphasized during the SARS epidemic [2–5, 42]. The public perception of fear was also demonstrated by the unprecedented increase in tweets expressing fear sentiments related to COVID-19 in January 2020 [43]. In Taiwan, the fear of COVID-19 may not be as high as that of SARS because the death rate and the death toll from COVID-19 infection in Taiwan were much lower; nonetheless, the impact on both ED censuses was similar. Therefore, the observation implied other reasons for this decline. During the COVID-19 pandemic, the emergence of telemedicine [44, 45] may have kept pediatric and lower-urgency patients away from attending the ED. However, this twenty-first century advance is unlikely to affect the decline of in our ED census, since telemedicine was not yet widely applicable in Taiwan [46]. The impact of influenza, SARS, and COVID-19 pandemics on ED visits is most pronounced in the pediatric and lower-urgency categories [2, 26, 47]. In contrast to influenza epidemics [47], the volume of the ED census during the SARS and COVID-19 epidemic decreased [2–5, 18–22, 26]. There is a high incidence of visits for upper respiratory diseases and infections, trauma, and the youngest age groups (< 5 years) in typical pediatric EDs [48, 49]. Respiratory tract infections account for the most common causes of pediatric treat-and-release ED visits [49]. Therefore, public health strategies that help to control the spread of flu-like illnesses should reduce ED visits. For example, lockdowns could lead to a reduction in the number of pediatric patients presenting an airborne infectious disease [50]. Coincidental with the COVID-19 pandemic, the case numbers of 14 airborne/droplet-transmitted infectious diseases decreased between January–October 2019 and January–October 2020 in Taiwan, with a reduction of 28.2% [51]. This might have contributed to a decrease in ED attendance, and even more so in the pediatric division. In addition to fear, a significant decrease in commonly transmitted respiratory diseases achieved by improving personal hygiene and protection may play an essential role in decreasing future ED attendance by those with relatively low acuity as well as pediatric patients.

The reduction in ambulatory visits and patients with lower acuity can be reasonably attributed to public fear of contracting the virus and a diminished transmission of respiratory diseases, but the numbers of higher acuity visits (levels I and II) also decreased to a similar degree. The unchanged ratio of patients within the top three acuity levels shows that the scale of our ED shrank in general compared to the pre-pandemic period. The ED divisions that often had patients with hospitalization requirements such as those with higher acuity, the elderly, and traumatic patients, also dramatically declined at first. Most of these patients likely arrive via non-ambulatory means, such as transport by EMS or transfer-in via ambulance. Since there was no decrease in EMS demand, we assume that, taking the decreased numbers of transfer-in patients into account, primary and secondary medical facilities also faced a reduction in the number of patients, which allowed them higher capacity to treat severe patients. We also assumed that among chronically critically ill patients, the choice of hospice at home or local nursing facilities increased. Where patients with higher acuity issues went warrants more comprehensive investigation. Unlike the decrease in the number of higher acuity visits, the number of patients with OHCA did not significantly change, which is different from areas devastated by the outbreak, where an increase in the percentage of EMS-attended deaths and OHCA was observed [52, 53]. It is possible that the number of impending death cases related to severe COVID-19 infections in our community were minimal and not unusual after the pandemic.

### Effects of COVID-19 on ED crowding in the initial and later phase of the pandemic

In the initial phase, despite a significant decline in total ED attendance except for the highest urgency (level I), the LOS of both discharged and hospitalized patients did not decrease. The D2d of acuity level II visits also did not shorten. These observations may imply that the novel disease, lacking information regarding its manifestations and transmission, may complicate the process of decision making and disposition made by physicians. Furthermore, the numbers of boarded beds in the hospital, usually an important factor affecting throughput and output, did not change significantly throughout the year, indicating that there was no pressure of loading reduction or even shut down in our hospital due to the impact of the pandemic. Finally, the percentage of adverse outcomes of our ED patients increased. The response capability of our medical team and administration seemed to be compromised early in the pandemic, but it could have been worse if COVID-19 cases surged simultaneously. A misjudgment could result in the implementation of cost-ineffective strategies that lead to a waste of human resources such as understaffing in screening and caring for patients with suspected COVID-19 infection with the pediatric department being overstaffed because of a drop in pediatric patient visits. A performance review of throughput and output at the ED will be essential, especially early in the pandemic, even in the low prevalence areas, to prevent the collapse of emergency medical care.

Intriguingly, a reverse change was noted in two throughput factors, D2d and LOS, in the later phase of the pandemic. Although it was short already, the D2d at our ED still demonstrated a significant decrease, making it an objective indicator of the performance and efficacy of ED staff. In addition, the LOS of hospitalized patients shortened significantly in all divisions, with a greater degree in the adult division, whereas the LOS of discharged patients shortened significantly only in the adult division. Although the reduction rate of discharge was higher than that of hospitalization, the reduction of LOS was significant among hospitalized patients rather than discharged patients. The divisions with longer LOS caused by overcrowding and more requirements for hospitalization seemed to benefit more from the overall reduction in patient numbers. Interestingly, the pediatric volume of patients decreased by nearly half, improving the LOS of hospitalized children rather than that of discharged children. This suggests that for divisions without overcrowding and prolonged LOS, a simple reduction in attendance, of which most cases are ambulatory and lower urgency, will not necessarily improve the LOS of discharged patients. The LOS affected less significantly was usually for categories approximately or less than 4 h. This outcome implies that patients who stayed longer hours at the ED contributed to crowding, which was previously mentioned [54]. ED physicians could pay more attention to patients who needed more time for work-up when unnecessary visits decreased. Except for the universal decrease in the influx of patients, the decrease in hospitalized patients from the ED contributed to relieving the pressure of admitted output. In the United States, despite a decrease in total encounters in many EDs, the LOS of patients was longer, thus attributing to surges in COVID-19 cases [55]. The overall improvement of crowding at our ED in a low-prevalence scenario also helped to reduce the incidence of URVs rather than IHCA and DAMA. Not only did physicians have extra time to evaluate medical problems, but the reluctance of patients to seek medical care could be another reason. The unchanged ratio of high urgency acuity patients may account for the stable incidence of IHCA and DAMA.

### Experience with COVID-19 in a low prevalence region

Our ED did not experience a surge in COVID-19 cases that would contribute to a longer LOS [21], there should theoretically be no notable difference when compared to the pre-pandemic period. However, there was a prolonged reduction in ED visits that could be attributed to an improved medical seeking behavior from an awareness of the importance of personal protection against COVID-19 rather than an alleviated fear of the pandemic because of the apparent drop in the number of cases with confirmed infection. Hence, we were able to improve the long-term crowding problems in non-trauma adults. Nevertheless, taking into account the gradual increase in ED attendance (Fig. 2), maintenance of a sustainable behavior in seeking emergency healthcare remains vital at the later stage of the pandemic to prevent a prolonged LOS similar to that before the pandemic (Fig. 5). An analysis of the change in characteristics of patient flow in an emergency setting throughout the pandemic in the current study may shed light on the role of reducing unnecessary visits in the alleviation of ED overcrowding. In this way, the implications of our study in a low COVID-19 prevalent area like Taiwan might provide a useful reference for health administrators and hospitals to improve the condition of ED overcrowding in the post-COVID-19 era.

### Comparison with SARS experience

Tracing the history of coronavirus pandemics, literature on the impact of the SARS epidemic in 2003 on the EDs at three tertiary hospitals from northern, central, and southern Taiwan were also reviewed [2–5]. The hospital from the south was our hospital (KSVGH). Compared with the pre-epidemic period, a month after the highest outbreak time in April 2003, the largest average declines in monthly visits were 51.6, 33.4, and 40%, respectively. The changes were also more significant in lower acuity and pediatric patients than in higher acuity and trauma patients. During the observation in 2003, one of these hospitals found that the total ED patient volume took a long time to return to the previous level [2]. Although the degrees of devastation caused by the SARS and COVID-19 epidemics are different, the immediate and long-term impact on visit numbers and characteristics of patients in the ED seem to be quite similar. Therefore, this phenomenon may repeat at the next novel coronavirus pandemic. The results of an analysis from a tertiary hospital in northern Taiwan could also support this hypothesis [56]. The historical experience from SARS to COVID-19 suggested that the unprecedented global coronavirus pandemic could be a rare but essential factor associated with the decision of non-urgent ED use that had not been previously mentioned in a systemic literature review [57].

### Limitations

Our study has several limitations. First, it is a single-center study that may not be generalizable or applicable to other emergency medicine institutions, since the hospital’s administrative policies could greatly affect the results, such as the LOS of patients. Nonetheless, the universal decrease in the need for medical care without increasing the crude death rate officially released by Taiwan’s government [16] was consistent with some of our findings. Second, since the cut-off point was set on January 31 instead of January 21, the date of the first reported case, comparing the changes in the initial epidemic phase between January and February 2020 was underestimated. Third, we did not adjust for multiple potentially confounding factors related to decreases in the ED flow other than the COVID-19 pandemic. Further analysis of different diagnostic categories may identify additional details about confounding factors. These factors may be influenced by decreased social distance and improved hand hygiene, resulting in a decrease in visits due to causes such as flu or gastroenteritis.

## Conclusions

The impacts of COVID-19 on ED attendance appeared similar regardless of disease prevalence in different regions in the early phase of the pandemic. Shortly after the initial phase in regions with a low prevalence, the decline in the ED influx volume, especially in pediatrics and in cases with lower acuity instead could have helped shorten the LOS of adult patients who usually experienced overcrowding at our ED. In contrast to devastated regions with a surge of COVID-19 cases, this outcome is attributable to a well-controlled epidemic and changes in medical behavior, and it highlights a potentially positive opportunity for public health administration worldwide to reduce unnecessary healthcare utilization in the post-COVID-19 era [58] and prepare emergency response protocols for probably the next coronavirus pandemic. The decreased patient flow to the ED that subsequently improved overcrowding in this low-COVID-19 prevalence region might help to evaluate the issue of emergency medical resource overuse.

## Supplementary Information

**Additional file 1: Supplemental Table 1.** Daily datasets of emergency department input, throughput, and output parameters from 2019 to 2020. DAMA: discharge against medical advice; IHCA: in-hospital cardiac arrest; LOS: length of stay; OHCA: out-of-hospital cardiac arrest.

**Additional file 2: Supplemental Table 2.** Monthly datasets of emergency department arrival modes, adverse outcomes, and admission numbers from February 2019 to December 2019, and from February 2020 to December 2020. ED: emergency department; EMS: emergency medical service; IHCA: in-hospital cardiac arrest; OHCA: out-of-hospital cardiac arrest.

## Data Availability

The datasets used during the current study are available from the corresponding author on reasonable request.

## Abbreviations

CI: Confidence interval
DAMA: Discharge against medical advice
ED: Emergency department
EMS: Emergency medicine service
IHCA: In-hospital cardiac arrest
LOS: Length of stay
MD: Mean difference
MERS: Middle East respiratory syndrome
OHCA: Out-of-hospital cardiac arrest
SARS: Severe acute respiratory syndrome
URVs: Unscheduled return visits
